# Reduction in allergen-specific IgE binding as measured by microarray: A possible surrogate marker for effects of specific immunotherapy

**DOI:** 10.1016/j.jaci.2015.02.034

**Published:** 2015-09

**Authors:** Eva Wollmann, Christian Lupinek, Michael Kundi, Regina Selb, Verena Niederberger, Rudolf Valenta

**Affiliations:** a Division of Immunopathology, Department of Pathophysiology and Allergy Research, Center for Pathophysiology, Infectiology and Immunology, Medical University of Vienna, Vienna, Austria; b Institute of Environmental Health, Center of Public Health, Medical University of Vienna, Vienna, Austria; c Department of Ear, Nose and Throat Diseases, Medical University of Vienna, Vienna, Austria

To the Editor:

*In vivo* provocation test methods (eg, skin testing and conjunctival provocation testing)^1^ are useful surrogates for clinical improvement, but the identification of *in vitro* markers for monitoring the effects of specific immunotherapy (SIT) has been a long-sought goal. It has been shown that allergen-specific blocking IgG antibodies inhibit allergen-induced mast cell and basophil degranulation as well as IgE-facilitated allergen presentation to T cells and is associated with a reduction of *in vivo* sensitivity.^1-4^ Cellular assays (eg, basophil activation assays and FAB assay)^5,6^ may allow uncovering and measuring the effects of allergen-specific blocking antibodies on the allergen-IgE interaction and to correlate *in vitro* results with clinical outcomes but are quite cumbersome.

We recently found that measurements of allergen-specific IgE levels performed in the presence of low allergen concentrations in the solid phase, for example, allergen microarrays, allow visualizing the inhibition of IgE binding in the presence of blocking IgG antibodies when allergen-specific blocking IgG antibodies are present.^7^ Therefore, it may be hypothesized that IgE measurements performed using low allergen concentrations such as in allergen microarrays may better reflect the *in vivo* patients' situation (ie, the patients' sensitivity).

We aimed to study the influence of SIT-induced allergen-specific IgG antibodies on IgE binding in microarray and CAP assays and to determine whether IgE levels measured by microarray are associated with clinical parameters. For this purpose, residual serum samples from a double-blind placebo-controlled immunotherapy trial performed in birch pollen–allergic patients with recombinant hypoallergenic Bet v 1 derivatives were analyzed.^2^ Sera were obtained before and immediately after treatment, shortly after the following birch season, and 1 year after starting the treatment (see timeline in Fig E1 in this article's Online Repository at www.jacionline.org) (placebo group, n = 27; recombinant Bet v 1 fragments, n = 17; recombinant Bet v 1 trimer, n = 21). A demographic characterization of the patients and their treatment (ie, cumulative doses administered and numbers of injections) can be found in Table E1 in this article's Online Repository at www.jacionline.org. Recombinant Bet v 1 fragments and trimer administered in this study are described in this article's Online Repository at www.jacionline.org.

Sera (Fig E1) were analyzed for Bet v 1–specific IgE and IgG levels by ImmunoCAP and ISAC, a multiallergen chip that contained 103 allergen-specific components to record kinetics of IgE and IgG responses (Thermo Fisher/Phadia AB, Uppsala, Sweden). Linear contrasts after ANOVA and correlations were calculated using Statistica 10.0 (StatSoft, Tulsa, Okla) and SPSS 22.0 (IBM, Armonk, NY).

In those patients who received active treatment and thus developed high levels of allergen-specific IgG, the detected Bet v 1–specific IgE antibodies differed strongly between ImmunoCAP and ISAC measurements in the sera obtained after but not before treatment. ImmunoCAP measurements showed significant increases in Bet v 1–specific IgE antibodies after treatment/before pollen season in both actively treated groups (Fig 1, *A*; see Table E2 in this article's Online Repository at www.jacionline.org), whereas detected Bet v 1–specific IgE levels decreased significantly when measured by ISAC compared to placebo-treated patients. Boosts of allergen-specific IgE production caused by seasonal allergen exposure were found in the “after-season” samples from all patients by ISAC and ImmunoCAP measurements, but, as earlier reported, increases were lower for actively treated patients than for placebo-treated patients^2^ (Fig 1, *B*). The decrease in Bet v 1–specific IgE measured by ISAC in the actively treated groups was associated with a strong increase in Bet v 1–specific IgG found by both CAP and ISAC measurements (Fig 1, *C* and *D*) and thus may be explained by blocking of Bet v 1–specific IgE binding by therapy-induced IgG in the ISAC. Immunization experiments performed with rBet v 1 fragments and trimer in animals following an immunization scheme close to the one used for this study showed that the trimer is more immunogenic than the fragments.^8^ This fits the observation that the trimer induced higher Bet v 1–specific IgG levels after vaccination as determined by quantitative CAP measurements (Fig 1, *C*, CAP: IgG increase comparing before treatment with after treatment; *P* < .05) than the fragments in the patients. Fragment-treated and trimer-treated patients had received comparable cumulative doses of the vaccines (Table E1). Therefore, the higher increase in Bet v 1–specific IgG in the trimer group was not due to different cumulative doses injected.

In contrast to the ISAC measurements, an increase in Bet v 1–specific IgE was found by CAP measurements because allergen is present in excess in the solid phase and therefore SIT-induced Bet v 1–specific IgE becomes visible. In fact, it is known that SIT also induces a rise in allergen-specific IgE.^9^ No relevant alterations in Bet v 1–specific IgG antibodies were observed for placebo-treated patients (Fig 1, *C* and *D*).

Rises in Bet v 1–specific IgE were observed for all groups as a result of seasonal allergen exposure after the pollen season and allergen-specific IgE then declined again 1 year after treatment before the next pollen season (Fig 1, *A* and *B*). Similar results of IgE and IgG antibody reactivities to Bet v 1–related pollen and plant food allergens (rAln g 1: alder; rCor a 1: hazel; rMal d 1: apple; rPru p 3: peach) were noted, but responses were lower than for Bet v 1, mirroring the degree of sequence similarity with Bet v 1 (Aln g 1 > Cor a 1 > Mal d 1 > Pru p 3) (see Figs E2 and E3 in this article's Online Repository at www.jacionline.org).

Next, we compared alterations in nasal allergen sensitivity as determined by active anterior rhinomanometry with changes in allergen-specific IgE levels measured by ISAC for those patients for whom nasal provocation data were available before treatment and after the pollen season (placebo, n = 22; rBet v 1 fragment, n = 12; rBet v 1 trimer, n = 16) (Fig 2). Results obtained 1 year after starting the treatment (Fig E1) were not analyzed because IgG levels had declined almost to baseline at this time point (Fig 1) and no differences between groups were found by nasal provocation.^10^ Nasal provocation was performed using increasing doses of natural birch pollen extract containing defined Bet v 1 concentrations. Changes in nasal allergen tolerance in the patients are represented by positive (increased nasal allergen tolerance) or negative (decreased nasal allergen tolerance) points, where 1 point indicates a 10-fold change to the results measured before treatment (Fig 2, y-axes).^10^ When changes in nasal sensitivity and allergen-specific IgE were plotted against each other, it became visible that patients with increases in Bet v 1–specific IgE without improvement or deterioration in nasal sensitivity were mainly found in the placebo group (Fig 2, placebo: right lower quarter) whereas patients with reduced Bet v 1–specific IgE were frequently observed in the actively treated group and often tolerated higher allergen doses during nasal provocation (Fig 2, left upper quarter; fragments: 25%, 3 of 12 patients, and trimer: 31.3%, 5 of 16 patients). Fig 2 shows that there is a significant correlation of the reduction in Bet v 1–specific IgE binding measured by ISAC with increased nasal allergen tolerance in the trimer-treated group (*r* = −0.620; *P* = .012). No significant correlation of the reduction in IgE binding to Bet v 1 determined by ISAC was found with increased nasal allergen tolerance in fragment-treated patients, which may be explained by the lower induction of allergen-specific IgG by fragments as compared to trimer (Fig 1, *C* and *D*).

Considering all treatment groups, decreases in IgE measured by ISAC seemed to be useful for the prediction of clinical improvement because we found a clinical improvement prediction of 90% (ie, 100% for placebo and trimer groups and 71% for the fragment group). This was not the case for increases in ΔIgE as measured by the ISAC, which was associated with a clinical worsening prediction of only 25% for the placebo group and 20% for trimer and fragment groups, respectively. A limitation of our study is that data were available only for a relatively small number of patients but our results indicate that decreases in allergen-specific IgE as measured on the chip are associated with reduced nasal allergen sensitivities. This effect was not at all observed when changes in allergen-specific IgE were measured under conditions of allergen excess by CAP because IgE levels increased in the placebo- and actively treated patients (see Fig E4 in this article's Online Repository at www.jacionline.org).

The results of our study thus indicate that allergen microarrays are useful to monitor the development of allergen-specific IgG responses during SIT, both against the allergen present in the SIT vaccine as well as against cross-reactive allergens. Moreover, the reduction in allergen-specific IgE binding measured by microarray analysis may be a useful surrogate marker for clinical effects of SIT, warranting more extensive prospective studies designed to analyze the association of IgE levels measured by microarray with results from *in vivo* allergen provocation and clinical end points.

## Figures and Tables

**Fig E1:**
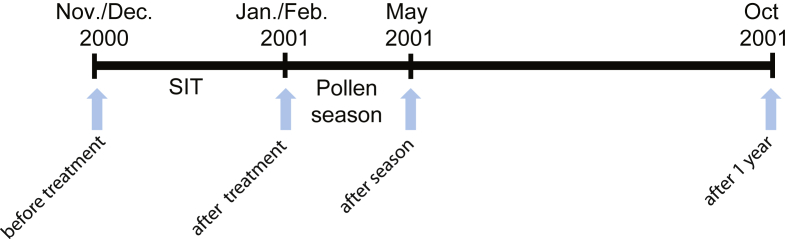
Timeline of the SIT study. *Blue arrows* indicate the time points when blood samples were taken (before treatment, after treatment, after season, after 1 year). Nasal provocation tests analyzed in this study were performed before treatment (November/December 2000) and after season (May 2001).

**Fig E2:**
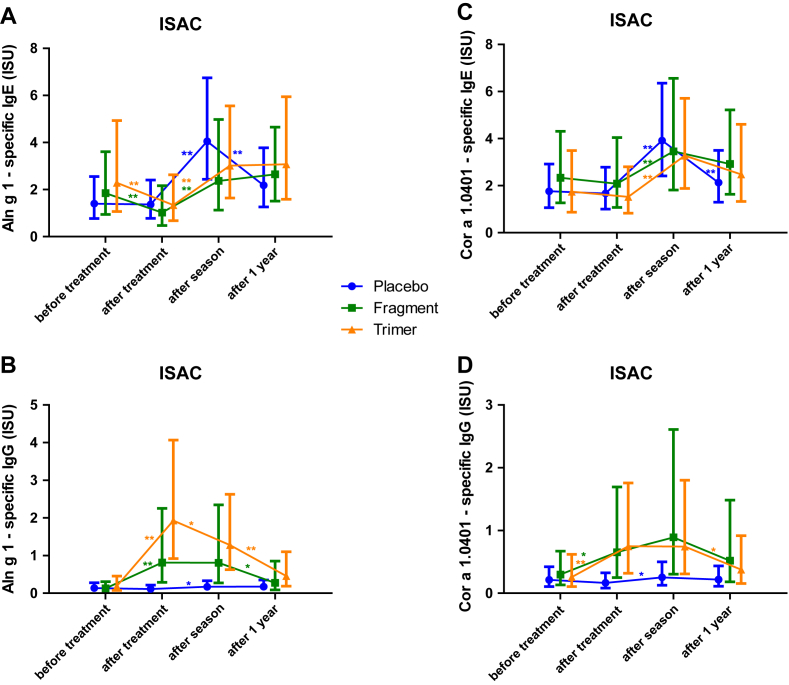
Courses of geometric means and 95% CIs of Aln g 1 (**A** and **B**) and Cor a 1 (**C** and **D**)-specific IgE and IgG levels. *P < .05 and **P < .01. *Asterisks* show the significance of changes within particular groups from the time point before treatment to after treatment, from after treatment to after season, and from after season to after 1 year.

**Fig E3:**
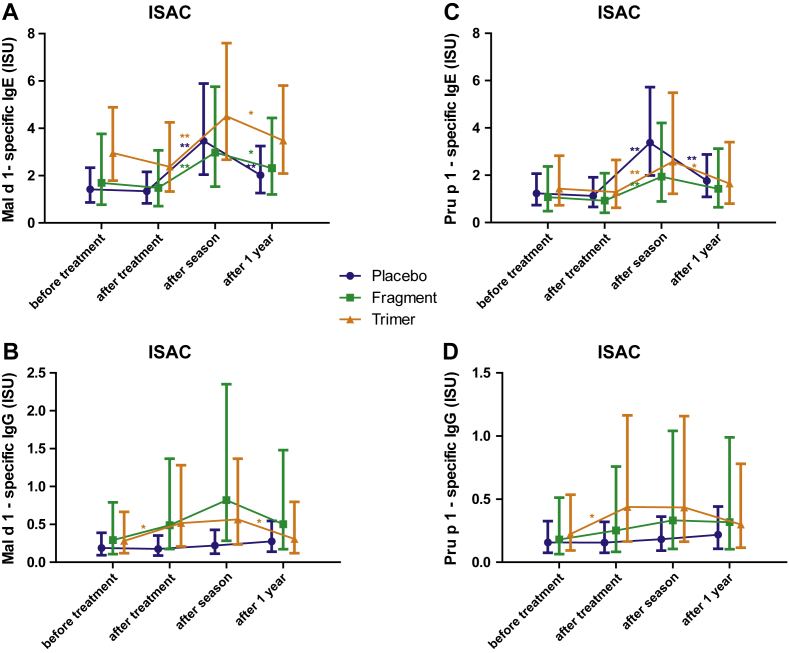
Course of geometric means and 95% CIs of Mal d 1 (**A** and **B**) or Pru p 1 (**C** and **D**)-specific IgE and IgG levels. *P < .05 and **P < .01. *Asterisks* show the significance of changes within particular groups from the time point before treatment to after treatment, from after treatment to after season, and from after season to after 1 year.

**Fig E4:**
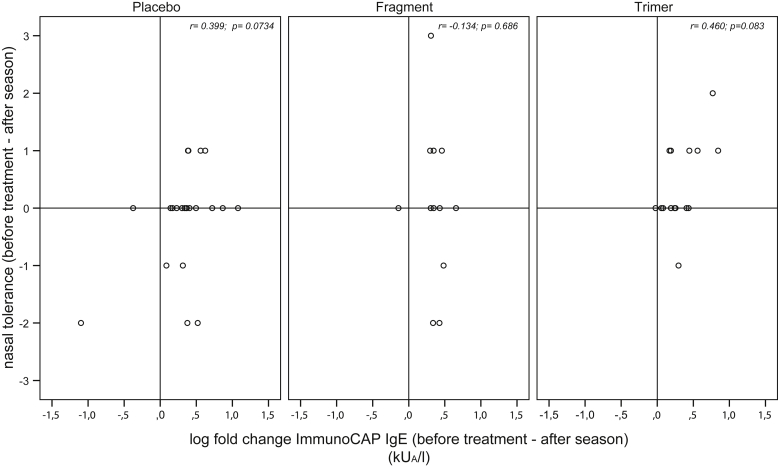
Association of alterations in Bet v 1–specific IgE levels determined by ImmunoCAP and nasal allergen tolerance. *Y-axes* (positive range) indicate increased allergen-specific tolerance, wherein 1-point increase indicates tolerance of a 10-fold higher allergen concentration and 1-point decrease indicates a 10-fold higher sensitivity. *X-axes* indicate the log fold changes of Bet v 1–specific IgE when comparing prestudy levels (ie, before treatment; see Fig 1 and Fig E1) to levels measured after the pollen season (ie, after season; see Fig 1 and Fig E1).

**Table E1:** Characterization of birch pollen–allergic patients

Characteristic	Treatment group (N = 65)		
Placebo (n = 27)	Bet v 1 fragments (n = 17)	Bet v 1 trimer (n = 21)
Sex (male/female)	33/32		
Age (y), median (range)	37 (23-55)	35 (23-58)	36 (24-57)
Cumulative dose (mg),	0	165	165
median (range)	(0)	(4-245)	(24-265)
Number of injections,	0	9	8
median (range)	(0-0)	(3-9)	(7-10)

**Table E2:** Geometric means and 95% CIs of allergen-specific IgE and IgG levels determined for Bet v 1 (ImmunoCAP, ISAC), Aln g 1 (ISAC), Cor a 1.0401 (ISAC), Mal d 1 (ISAC), and Pru p 1 (ISAC)

Measurement	Geometric mean, 95% CI (low-high)				
Group	Before treatment	After treatment	After season	After 1 y
For Bet v 1					
Immuno CAP IgE	Placebo (n = 27)	11.1 (7.6-16.1)	12.2 (8.2-18.1)	24.7 (15.4-39.9)	19.6 (12.8-29.9)
	Fragment (n = 17)	18.4 (11.4-29.8)	29.1 (17.6-48.0)	42.5 (28.4-63.8)	25.8 (13.7-48.5)
	Trimer (n = 21)	20.3 (13.6-30.2)	34.2 (24.0-48.8)	43.2 (31.1-60.0)	30.0 (21.2-42.3)
Immuno CAP IgG	Placebo (n = 27)	2.9 (2.4-3.5)	3.0 (2.3-3.8)	3.4 (2.8-4.3)	3.4 (2.8-4.1)
	Fragment (n = 17)	2.9 (2.5-3.4)	6.0 (4.3-8.4)	4.7 (3.6-6.3)	3.8 (3.1-4.7)
	Trimer (n = 21)	3.3 (2.7-4.1)	10.3 (7.4-14.3)	7.9 (5.5-11.6)	5.2 (4.0-6.8)
ISAC IgE	Placebo (n = 27)	9.3 (6.5-13.5)	8.9 (6.2-12.7)	20.1 (14.3-28.5)	11.6 (8.0-16.8)
	Fragment (n = 17)	10.5 (6.2-17.8)	4.9 (2.7-9.0)	10.6 (5.5-20.3)	11.9 (7.6-18.5)
	Trimer (n = 21)	11.8 (7.8-18.1)	4.3 (2.7-6.6)	11.3 (7.3-17.4)	10.1 (6.7-15.3)
ISAC IgG	Placebo (n = 27)	1.1 (0.8-1.7)	0.9 (0.5-1.3)	0.9 (0.5-1.5)	0.9 (0.6-1.5)
	Fragment (n = 17)	1.1 (0.8-1.7)	3.1 (1.7-5.8)	2.9 (1.4-6.0)	2.2 (1.2-4.1)
	Trimer (n = 21)	1.2 (0.7-1.9)	5.1 (3.0-8.6)	4.1 (2.4-6.7)	1.5 (0.9-2.7)
Aln g 1					
ISAC IgE	Placebo (n = 27)	1.4 (0.8-2.6)	1.4 (0.8-2.4)	4.1 (2.4-6.7)	2.2 (1.3-3.8)
	Fragment (n = 17)	1.9 (0.9-3.6)	1.0 (0.5-2.2)	2.4 (1.1-5.0)	2.7 (1.5-4.7)
	Trimer (n = 21)	2.3 (1.1-4.9)	1.3 (0.7-2.6)	3.0 (1.6-5.6)	3.0 (1.6-5.9)
ISAC IgG	Placebo (n = 27)	0.1 (0.1-0.3)	0.1 (0.1-0.2)	0.2 (0.1-0.3)	0.2 (0.1-0.4)
	Fragment (n = 17)	0.1 (0.1-0.3)	0.8 (0.3-2.3)	0.8 (0.3-2.3)	0.3 (0.1-0.9)
	Trimer (n = 21)	0.2 (0.1-0.5)	1.9 (0.9-4.1)	1.3 (0.6-2.6)	0.5 (0.2-1.1)
Cor a 1.0401					
ISAC IgE	Placebo (n = 27)	1.8 (1.1-2.9)	1.7 (1.0-2.8)	3.9 (2.4-6.4)	2.1 (1.3-3.5)
	Fragment (n = 17)	2.3 (1.3-4.3)	2.1 (1.1-4.0)	3.5 (1.8-6.6)	2.9 (1.6-5.2)
	Trimer (n = 21)	1.7 (0.9-3.5)	1.5 (0.8-2.8)	3.3 (1.9-5.7)	2.5 (1.3-4.6)
ISAC IgG	Placebo (n = 27)	0.2 (0.1-0.4)	0.2 (0.1-0.3)	0.3 (0.5-0.1)	0.2 (0.4-0.1)
	Fragment (n = 17)	0.3 (0.1-0.7)	0.7 (0.3-1.7)	0.9 (0.3-2.6)	0.5 (0.2-1.5)
	Trimer (n = 21)	0.3 (0.1-0.6)	0.7 (0.3-1.8)	0.7 (0.3-1.8)	0.4 (0.2-0.9)
Mal d 1					
ISAC IgE	Placebo (n = 27)	1.4 (0.9-2.3)	1.3 (0.8-2.2)	3.5 (2.0-5.9)	2.0 (1.3-3.3)
	Fragment (n = 17)	1.7 (0.8-3.8)	1.5 (0.7-3.1)	3.0 (1.5-5.8)	2.3 (1.2-4.4)
	Trimer (n = 21)	3.0 (1.8-4.9)	2.4 (1.3-4.3)	4.5 (2.7-7.6)	3.5 (2.1-5.8)
ISAC IgG	Placebo (n = 27)	0.2 (0.1-0.4)	0.2 (0.1-0.4)	0.2 (0.1-0.4)	0.3 (0.5-0.1)
	Fragment (n = 17)	0.3 (0.1-0.8)	0.5 (0.2-1.4)	0.8 (0.3-2.4)	0.5 (0.2-1.5)
	Trimer (n = 21)	0.3 (0.1-0.7)	0.5 (0.2-1.3)	0.6 (0.2-1.4)	0.3 (0.1-0.8)
Pru p 1					
ISAC IgE	Placebo (n = 27)	1.2 (0.7-2.1)	1.1 (0.7-1.9)	3.4 (2.0-5.7)	1.8 (1.1-2.9)
	Fragment (n = 17)	1.1 (0.5-2.4)	0.9 (0.4-2.1)	1.9 (0.9-4.2)	1.4 (0.7-3.1)
	Trimer (n = 21)	1.4 (0.7-2.8)	1.3 (0.6-2.6)	2.6 (1.2-5.5)	1.6 (0.8-3.4)
ISAC IgG	Placebo (n = 27)	0.2 (0.1-0.3)	0.2 (0.1-0.3)	0.2 (0.1-0.4)	0.2 (0.1-0.4)
	Fragment (n = 17)	0.2 (0.1-0.5)	0.3 (0.1-0.8)	0.3 (0.1-1.0)	0.3 (0.1-1.0)
	Trimer (n = 21)	0.2 (0.1-0.5)	0.4 (0.2-1.2)	0.4 (0.2-1.2)	0.3 (0.1-0.8)

**Fig 1:**
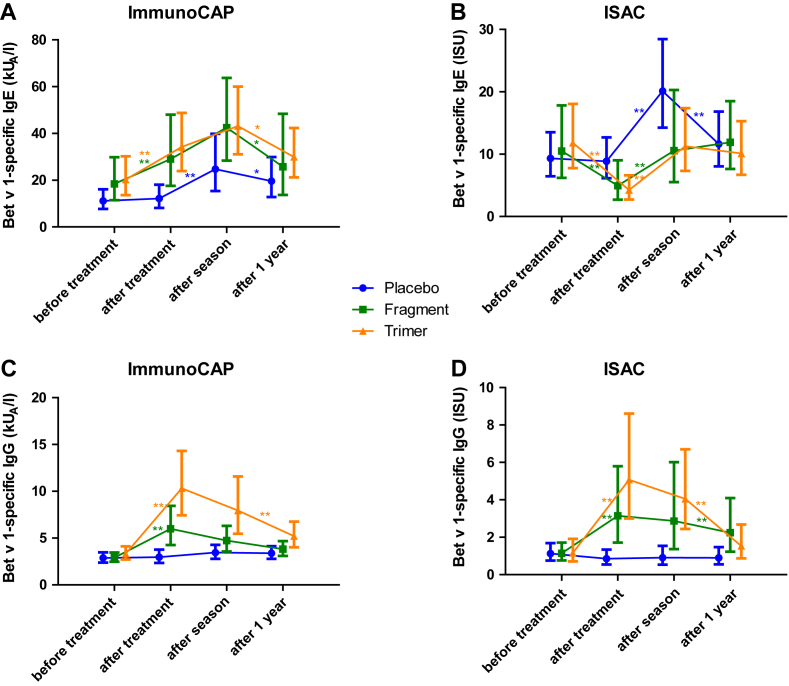
Courses of geometric means and 95% CIs of Bet v 1–specific IgE (**A**: ImmunoCAP; **B**: ISAC) and IgG (**C**: ImmunoCAP; **D**: ISAC) levels *(y-axes)* over the study period *(x-axes)* for placebo- *(blue)*, fragment- *(green)*, and trimer-treated patients *(orange)*. *P < .05 and **P < .01. *Asterisks* show the significance of changes within particular groups from the time point before treatment to after treatment, from after treatment to after season, and from after season to after 1 year. *ISU*, ISAC Standardized Unit.

**Fig 2:**
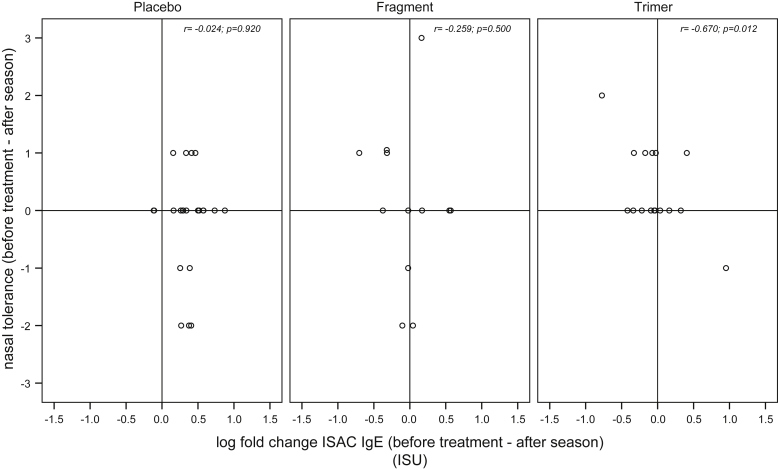
Association of alterations in Bet v 1–specific IgE levels determined by ISAC and nasal allergen tolerance. *Y-axes* (positive range) indicate increased allergen-specific tolerance, wherein 1-point increase indicates tolerance of a 10-fold higher allergen concentration and 1-point decrease indicates a 10-fold higher sensitivity. *X-axes* indicate the log fold changes of Bet v 1–specific IgE when comparing prestudy levels (ie, before treatment; see Fig 1 and Fig E1) to levels after the pollen season (ie, after season; see Fig 1 and Fig E1). *ISU*, ISAC Standardized Unit.

## Methods

### Serum samples

Serum samples were residual samples from an immunotherapy trial conducted with recombinant hypoallergenic Bet v 1 derivatives.^E1^ The study began in November/December 2000, and the treatment course finished in January/February 2001 before the birch pollen season, which lasted from February to May 2001. The time point after 1 year described the follow-up visit in October 2001. Eight up-dosing injections of fragment, trimer, or placebo were administered (1, 2, 4, 8, 10, and 20 μg of protein) in 1 or 2 weekly intervals as a preseasonal treatment. After reaching the maximal dose of 80 μg in the active groups, treatment was continued at 4 weekly intervals until the beginning of the flowering season (Fig E1).

### Administered immunotherapy preparations

Two recombinant Bet v 1 fragments representing amino acids 1 to 74 and 75 to 160 of the Bet v 1 allergen, respectively, were expressed in *Escherichia coli*. These fragments showed almost no allergenic activity and a more than 100-fold reduced IgE-binding capacity and allergenic activity compared with the complete allergen molecule.^E2^ The recombinant Bet v 1 trimer protein consisted of 3 covalently linked copies of Bet v 1. Like the fragments, it showed reduced allergenic activity.^E3^ Despite the lack of allergenic activity, both types of derivatives induced after immunization robust production of IgG antibodies in animals that also bound to the Bet v 1 allergen and inhibited allergic patients' IgE recognition of the allergen as well as allergen-induced basophil degranulation.

### Nasal provocation

Nasal provocation testing in the study was conducted with natural birch pollen extract containing defined concentrations of Bet v 1. Provocation and rhinomanometry measurements by active anterior rhinomanometry (Allergopharma Rhinomanometer, Reinbek, Germany) were performed by a trained investigator before treatment, after pollen seasons, and 1 year after the study started. Baseline levels (nasal flow and resistance) were established with 0.9% sodium chloride solution. Then, patients received intranasally (see below) increasing doses of birch pollen extract solution (0.0064, 0.064, 0.64, and 6.4 μg/mL), and 15 to 20 minutes postallergen application changes in nasal parameters were determined. The evaluation was performed exactly as described by Reisinger et al.^E4^

### Measurement of allergen-specific IgE and IgG

ImmunoCAP measurements were done according to the manufacturer's instructions. ISAC chips were washed and 25 μL of the undiluted serum samples was applied on the chip and incubated for 120 minutes at room temperature. For allergen-specific IgG detection, samples were diluted 1:50 in PBS + 0.5% Tween 20. After washing, the chips were dried and incubated with 25 μL of fluorescence-labeled anti-IgE detection antibody or anti-IgG detection antibody, respectively, for 60 minutes at room temperature. Chips were analyzed using the Laser Scan Confocal microarray reader (LuxScan 10K/A; Capital-Bio, Beijing, China). Results were evaluated using Phadia Microarray Image Analysis software and reported in semi-quantitative ISAC Standardized Units (ISU) with a cutoff of 0.3 ISU as recommended by the manufacturer.

Calibration was performed with a commercial calibrator serum pool reacting with a wide range of allergens with high and low IgE levels. For these allergens ImmunoCAP measurements were performed. According to manufacturer's specifications, the ImmunoCAP-specific IgE calibrators can be traced to the 2nd International Reference Preparation 75/502 of Human Serum Immunoglobulin E from the World Health Organization. The same reference sera are afterwards analyzed by using the microarray technology. Resulting fluorescence values of the very same allergens tested by ImmunoCAP are correlated with results of these samples acquired by ImmunoCAP testing and correlation curves are then established. Calibration is performed in every test run to maintain a consistent and reliable data generation.

This provides an indirect link of the ISU IgE antibody results obtained by microarray testing with the World Health Organization International Reference Preparation 75/502 IgE. Likewise, IgG calibration is achieved with the help of reference sera, which are run in parallel for a broad panel of allergens on IgG ImmunoCAPs and on the ISAC.^E5^

### Statistical tests

Linear contrasts after ANOVA and correlations were calculated using Statistica 10.0 (StatSoft) and SPSS 22.0 (IBM). Table E2 provides geometric means for IgE and IgG measurements performed by ImmunoCAP and ISAC for Bet v 1 and for IgE and IgG measurements done by ISAC for Aln g 1, Cor a 1.0401, Mal d 1, and Pru p 1.
